# Supplementary material for: Comparison of development of step-kinematics of assisted 60 m sprints with different pulling forces between experienced male and female sprinters
Source: PLoS One. 2021 Jul 27;16(7):e0255302. doi: 10.1371/journal.pone.0255302 (PMC8315524; doi:10.1371/journal.pone.0255302)
Supplement: S1 Data — (PDF) [file pone.0255302.s001.pdf]

subjectgroup	height	weight	age	PB	normal	3kg
1	195	73	18	12.10	7.66	7.55
1	170	68	27	10.27	6.72	6.53
1	185	83	33	11.15	7.27	7.25
1	180	80	24	10.65	7.04	6.74
1	186	80	18	11.46	7.33	7.36
1	183	84	21	10.78	7.08	7.05
1	185	74	16	11.82	7.39	7.22
1	180	70	16	11.35	7.6	7.38
1	186	78	30	10.64	6.87	6.8
1	166	58	18	11.10	6.85	6.69
1	183	75	24	11.12	7.2	7.08
1	184	69	17	11.91	7.46	7.27
2	175	64	21	11.89	7.76	7.395
2	160	55	19	12.46	8.18	7.63
2	168	68	27	11.74	7.65	7.61
2	176	63	16	13.07	8.33	8.04
2	168	69	18	13.36	8.32	8.19
2	178	58	20	13.11	8.4	7.88
2	158	52	18	12.62	8.22	7.9
2	170	65	20	12.97	8.02	7.86
2	158	55	22	13.07	8.35	8.15
2	164	58	20	11.94	8.04	7.82
2	158	56	21	13.54	8.74	8.35
2	168	58	24	13.01	8.02	7.86

4kg	5kg	velnor5	velnor10	velnor15	velnor20	velnor25
7.46	7.45	5.52	6.42	7.12	7.57	7.94
6.42	6.33	5.29	6.87	7.83	8.4	8.87
7.12	6.92	4.85	6.22	7.05	7.75	8.18
6.85	6.83	5.28	6.64	7.52	8.07	8.52
7.27	7.21	5.42	6.61	7.19	7.66	8.17
6.87	6.7	5.48	6.73	7.34	7.85	8.4
7.14	6.93	5.22	6.64	7.28	7.79	8.22
7.32	7.23	4.49	6.61	7.18	7.64	8.14
6.75	6.64	5.57	6.73	7.65	8.2	8.66
6.55	6.52	5.39	6.94	7.55	8.23	8.65
		5.37	6.63	7.34	8.1	8.55
7.29	7.15	4.13	6.47	7.49	8.02	8.41
7.35	7.13	3.81	5.86	6.83	7.31	7.68
7.63	7.34	3.82	5.86	6.76	7.19	7.61
7.36	7.31	4.1	6.22	7.23	7.67	8.01
7.9	7.79	4.16	5.83	6.53	6.92	7.23
8.01	7.86	3.9	5.49	6.3	6.88	7.29
7.55	7.49	3.51	6.47	6.69	6.92	7.39
7.81	7.61	4.44	6.07	6.64	7.07	7.48
7.83	7.67	3.94	5.44	6.45	7.19	7.69
8.05	7.98	4.32	5.74	6.42	6.92	7.26
7.74	7.6	3.99	6.69	7.21	7.5	7.76
8.27	8.18					
7.59	7.47	3.94	5.44	6.45	7.19	7.69

velnor30	velnor35	velnor40	velnor45	velnor50	velnor55	velnor60
8.27	8.53	8.75	8.91	9.04	9.1	9.16
9.35	9.64	9.79	9.95	10.07	10.13	10.17
8.52	8.8	9.01	9.17	9.28	9.35	9.38
9.02	9.3	9.46	9.65	9.79	9.88	9.94
8.47	8.71	8.9	9	9.1	9.18	9.22
8.73	9	9.2	9.36	9.48	9.53	9.58
8.53	8.78	8.95	9.07	9.13	9.16	9.16
8.44	8.66	8.84	8.92	9.02	9.07	9.1
9.03	9.24	9.49	9.67	9.75	9.8	9.84
8.98	9.34	9.53	9.64	9.75	9.82	9.85
8.93	9.22	9.44	9.61	9.72	9.78	9.81
8.73	8.97	9.17	9.27	9.38	9.46	9.49
8.06	8.33	8.49	8.61	8.71	8.78	8.84
7.85	8.02	8.16	8.25	8.32	8.34	8.34
8.31	8.49	8.61	8.73	8.78	8.82	8.83
7.54	7.77	7.89	7.98	8.05	8.08	8.1
7.51	7.68	7.81	7.9	7.97	8.01	8.05
7.83	8.19	8.4	0.00	0.00	0.00	0.00
7.76	7.89	7.98	8.06	8.1	8.12	8.14
7.94	8.1	8.24	8.32	8.39	8.45	8.51
7.47	7.58	7.64	7.69	7.71	7.73	7.75
7.98	8.22	8.46	8.65	8.81	8.84	8.78
7.94	8.1	8.24	8.32	8.39	8.45	8.51

velnor65	velnor70	velnor75	velnor80	velnor85	velnor90	velnor95
9.19	9.19	9.16	9.11	9.04	8.95	8.88
10.18	10.17	10.16	10.15	10.15	10.16	10.2
9.39	9.39	9.35	9.31	9.28	9.24	9.22
9.96	9.97	9.96	9.94	9.91	9.88	9.86
9.24	9.25	9.25	9.24	9.24	9.25	9.26
9.59	9.58	9.56	9.52	9.47	9.41	9.38
9.15	9.12	9.1	9.09	9.09	9.13	9.2
9.11	9.1	9.08	9.06	9.04	9.03	9.04
9.84	9.81	9.75	9.66	9.59	9.52	9.39
9.85	9.82	9.77	9.7	9.63	9.55	9.5
9.8	9.77	9.73	9.66	9.58	9.49	9.4
9.52	9.54	9.54	9.54	9.53	9.51	9.44
8.88	8.92	8.95	8.96	8.96	8.89	8.83
8.32	8.3	8.27	8.25	8.23	8.18	8.11
8.84	8.84	8.84	8.82	8.78	8.69	8.55
8.1	8.08	8.07	8.04	8.02	8	7.95
8.07	8.08	8.09	8.08	8.05	8.01	7.93
0.00	0.00	0.00	0.00	0.00	0.00	0.00
8.15	8.16	8.18	8.18	8.18	8.15	8.1
8.56	8.58	8.58	8.52	8.42	8.34	8.21
7.76	7.78	7.8	7.81	7.8	7.77	7.68
8.67	8.53	8.41	8.37	8.49	8.72	9.08
8.56	8.58	8.58	8.52	8.42	8.34	8.21

velnor100	freqnor5	freqnor10	freqnor15	freqnor20	freqnor25	freqnor30
8.76	3.52	3.89	4.04	4.08	4.08	4.08
10.28	4.33	4.6	4.83	5	4.96	4.77
9.21	4.15	4.89	4.37	4.65	4	4.59
9.85	4.53	4.46	4.72	4.65	4.6	4.6
9.3	4.12	4.21	4.35	4.36	4.31	4.08
9.32	4.27	4.27	4.35	4.4	4.6	4.35
9.35	3.95	4.17	4.35	4.21	4.26	4.26
9.07	4.1	4.17	4.35	4.17	4.4	4.44
9.27	4	4.13	4.17	4.26	4.17	4.35
9.42	4.05	4.76	4.6	4.66	4.55	4.71
9.32	4.09	3.77	4.4	4.65	4.88	4.67
9.24	3.37	3.87	4.11	4.18	4.2	4.4
8.62	4.43	4.55	4.44	4.47	4.54	4.45
7.85	4.62	3.74	3.91	4	4.18	4.51
8.23	4.77	4.67	4.3	3.93	4.98	4.51
7.85	3.11	4.08	4.17	4.1	4.12	4.27
7.8	3.28	3.84	3.86	3.97	4.04	3.97
0.00	3.19	3.64	3.9	3.83	4.14	4.18
7.97	4.71	3.91	4.07	4.28	4.3	4.47
8.15	3.47	3.99	4.04	4.18	4.21	4.22
7.52	4.29	4.24	4.48	4.47	4.52	4.55
9.63	3.66	4.43	4.5	4.56	4.51	4.72
8.15	3.47	3.99	4.04	4.18	4.21	4.22

freqnor35	freqnor40	freqnor45	freqnor50	freqnor55	freqnor60	freqnor65
4.12	4.08	4.17	4	4.17	4.12	4.17
4.35	5.26	4.71	4.65	4.89	5.07	4.44
5.13	4.6	4.17	4.57	5.13	4.71	4.08
4.55	4.76	4.6	4.65	4.65	5	4.79
4.37	4.44	4.17	4.45	4.44	4.26	4.26
4.55	4.65	4.44	4.65	4.35	4.45	4.55
4.26	4.26	4.17	4.3	4.35	4.17	4.21
4.26	4.44	4.26	4.4	4.08	4.3	4.26
4.08	4.08	4.08	3.92	4.26	4.22	4.17
4.76	4.65	4.55	4.65	4.76	4.76	4.7
4.08	4.6	5.13	4.71	4.08	5.13	4.67
4.09	4.42	4.15	4.29	4.54	4.08	4.41
4.54	4.33	4.53	4.53	4.54	4.34	4.39
4.07	4.06	4.49	4.04	4.35	4	4.22
4.62	4.48	4.64	4.54	4.59	4.65	4.76
4.14	4.24	4.2	4.07	4.21	4.35	4.17
4.1	3.98	4.11	3.99	4.07	4.09	4.12
3.96	4.16	0.00	0.00	0.00	0.00	0.00
4.53	4.58	4.49	4.44	4.54	4.55	4.25
4.42	4.07	4.34	4.29	4.25	4.12	4.34
4.41	4.69	4.65	4.71	4.44	4.61	4.64
4.5	4.61	4.57	4.59	4.66	4.46	4.57
4.42	4.07	4.34	4.29	4.25	4.12	4.34

freqnor70	freqnor75	freqnor80	freqnor85	freqnor90	freqnor95	freqnor100
4.26	4.17	4.08	4.21	4.17	4.26	3.96
5.13	4.69	4.55	4.84	5	4.71	3.7
5	5.13	4.58	4.17	4.54	5.26	4.4
4.17	5.26	4.52	4.55	4.55	5	4.52
4.65	4.26	4.08	4.33	4.55	4	4.04
4.55	4.44	4.55	4.36	4.35	4.35	3.85
4.17	4.21	4.26	4.17	4.1	4.44	3.85
4.35	4.17	4.35	4.12	4.26	3.92	3.91
4.17	4.26	3.92	4.35	3.77	3.91	4
4.55	4.71	4.76	4.76	4.65	4.76	4.38
4	5.13	4.52	3.92	4.59	4.55	3.76
4.08	4.35	4.26	4.35	4.22	3.87	3.98
4.64	4.25	4.17	4.65	4.36	3.95	3.98
4.26	4.17	4.09	4.35	4.66	4.02	3.63
4.08	5.03	4.6	4.56	4.68	4.12	4.17
4.09	4.13	4.09	4.17	4.13	4.01	4.03
4.08	4.04	4.09	4.01	4.09	4.1	4.09
0.00	0.00	0.00	0.00	0.00	0.00	0.00
4.49	4.5	4.26	4.47	4.45	4.77	3.48
4.17	4.17	4.1	4.46	4.02	4.11	4.08
4.59	4.49	4.55	4.61	4.68	4.59	4.14
4.52	4.66	4.59	4.51	4.49	4.3	4.13
4.17	4.17	4.1	4.46	4.02	4.11	4.08

lengnor5	lengnor10	lengnor15	lengnor20	lengnor25	lengnor30	lengnor35
1.57	1.65	1.76	1.85	1.95	2.03	2.07
1.23	1.5	1.63	1.68	1.79	1.96	2.22
1.18	1.27	1.62	1.68	2.04	1.9	1.72
1.18	1.5	1.6	1.73	1.85	1.96	2.05
1.32	1.57	1.65	1.76	1.9	2.08	2
1.28	1.58	1.69	1.79	1.83	2.01	1.98
1.32	1.59	1.68	1.85	1.93	2.01	2.06
1.07	1.59	1.65	1.84	1.85	1.9	2.04
1.39	1.63	1.83	1.93	2.08	2.08	2.26
1.32	1.46	1.64	1.77	1.9	1.91	1.96
1.28	1.55	1.69	1.8	1.92	1.98	2.05
1.22	1.68	1.82	1.92	2	1.98	2.19
0.87	1.3	1.56	1.64	1.69	1.81	1.85
0.9	1.57	1.73	1.81	1.83	1.74	1.98
0.72	1.35	1.68	1.95	1.62	1.84	1.83
1.44	1.43	1.57	1.69	1.76	1.77	1.88
1.19	1.43	1.63	1.73	1.81	1.89	1.87
1.09	1.78	1.72	1.81	1.79	1.87	2.07
1.11	1.55	1.63	1.65	1.74	1.74	1.74
1.13	1.37	1.6	1.72	1.83	1.88	1.83
1.04	1.36	1.44	1.55	1.61	1.64	1.72
1.04	1.51	1.61	1.64	1.72	1.69	1.83
1.13	1.37	1.6	1.72	1.83	1.88	1.83

lengnor40	lengnor45	lengnor50	lengnor55	lengnor60	lengnor65	lengnor70
2.14	2.14	2.26	2.19	2.22	2.21	2.16
1.86	2.11	2.17	2.08	2.03	2.29	1.98
1.98	2.2	2.04	1.82	2.02	2.3	1.88
1.99	2.1	2.1	2.12	1.99	2.09	2.39
2	2.16	2.05	2.07	2.17	2.17	1.99
1.98	2.11	2.04	2.19	2.15	2.11	2.11
2.1	2.18	2.12	2.11	2.2	2.17	2.19
1.99	2.1	2.05	2.22	2.12	2.14	2.09
2.32	2.37	2.49	2.3	2.34	2.36	2.36
2.05	2.12	2.1	2.06	2.07	2.15	2.16
2.04	2.17	2.15	2.11	2.14	2.21	2.14
2.07	2.24	2.19	2.09	2.33	2.17	2.34
1.96	1.9	1.92	1.94	2.04	2.02	1.92
2.01	1.84	2.06	1.92	2.08	1.98	1.95
1.92	1.88	1.94	1.92	1.9	1.86	2.17
1.86	1.9	1.97	1.92	1.86	1.94	1.98
1.96	1.92	2	1.97	1.97	1.96	1.98
2.02	0.00	0.00	0.00	0.00	0.00	0.00
1.74	1.8	1.82	1.79	1.79	1.92	1.82
2.03	1.92	1.95	1.99	2.07	1.97	2.06
1.63	1.66	1.64	1.74	1.69	1.67	1.69
1.83	1.89	1.92	1.9	1.97	1.9	1.89
2.03	1.92	1.95	1.99	2.07	1.97	2.06

lengnor75	lengnor80	lengnor85	lengnor90	lengnor95	lengnor100	flightnor5
2.2	2.23	2.15	2.15	2.09	2.21	65
2.19	2.23	2.11	2.03	2.17	2.78	70
1.82	2.05	2.23	2.06	1.75	2.12	90
1.89	2.21	2.18	2.17	1.97	2.19	67.5
2.17	2.27	2.15	2.03	2.32	2.33	96.67
2.15	2.1	2.18	2.17	2.16	2.42	65
2.16	2.14	2.18	2.24	2.07	2.45	91.67
2.18	2.08	2.19	2.12	2.31	2.34	91.67
2.29	2.46	2.21	2.52	2.42	2.32	92.5
2.08	2.04	2.02	2.05	1.99	2.17	60
2.12	2.19	2.15	2.15	2.15	2.33	70
2.19	2.24	2.19	2.25	2.44	2.33	103.33
2.1	2.15	1.92	2.04	2.24	2.17	127.75
1.98	2.02	1.89	1.76	2.02	2.16	88.75
1.77	1.92	1.93	1.86	2.07	1.97	129
1.95	1.97	1.92	1.94	1.98	1.95	149
2	1.98	2.01	1.96	1.93	1.93	131.67
0.00	0.00	0.00	0.00	0.00	0.00	113.75
1.84	1.92	1.84	1.85	1.7	1.69	98.75
2.06	2.08	1.89	2.07	2	1.99	106.67
1.74	1.72	1.69	1.66	1.67	1.83	112.25
1.8	1.82	1.88	1.94	2.12	2.33	96.25
2.06	2.08	1.89	2.07	2	1.99	106.67

flightnor10	flightnor15	flightnor20	flightnor25	flightnor30	flightnor35	flightnor40
95	112.5	115	122.5	125	127.5	130
87.5	95	90	100	107.5	130	100
87.5	100	105	130	112.5	95	115
75	75	85	95	105	110	100
102.5	95	105	115	125	112.5	115
85	100	95	92.5	110	110	105
107.5	105	112.5	120	120	120	122.5
102.5	95	120	112.5	105	120	115
107.5	110	110	120	120	125	135
85	97.5	102.5	115	105	100	110
85	105	95	80	100	130	110
135	137.5	140	150	150	152.5	140
103.33	135	120	135	132.5	142.5	135
141.67	110	117.5	122.5	130	135	140
111.67	130	105	137.5	135	140	140
110	122.5	130	125	125	137.5	125
127.5	137.5	137.5	145	145	142.5	150
120	125	137.5	127.5	135	137.5	140
102.5	120	122.5	130	130	120	122.5
105	110	130	125	125	125	130
105	112.5	120	120	130	125	125
117.5	122.5	127.5	132.5	125	137.5	135
105	110	130	125	125	125	130

flightnor45	flightnor50	flightnor55	flightnor60	flightnor65	flightnor70	flightnor75
125	130	125	122.5	125	120	122.5
117.5	125	110	102.5	90	95	125
130	120	100	115	140	105	100
115	115	115	100	110	115	115
130	112.5	110	125	125	105	120
115	105	120	117.5	110	105	120
115	117.5	120	120	125	130	125
120	120	135	132.5	125	125	135
135	145	135	132.5	135	135	130
110	110	110	107.5	115	105	102.5
100	110	135	90	110	140	90
165	137.5	135	170	150	150	152.5
120	125	130	135	137.5	150	152.5
122.5	132.5	125	140	145	135	140
145	130	140	125	135	110	145
135	140	142.5	125	137.5	140	135
140	160	145	140	145	150	147.5
160	145	147.5	130	142.5	145	135
125	130	125	122.5	135	127.5	142.5
110	140	150	127.5	110	130	125
122.5	120	130	125	130	125	122.5
135	135	130	145	140	145	140
110	140	150	127.5	110	130	125

flightnor80	flightnor85	flightnor90	flightnor95	flightnor100	contactnor5	contactnor10
130	122.5	125	120	135	220	162.5
130	117.5	105	117.5	165	166.67	130
120	140	127.5	100	127.5	153.33	117.5
127.5	125	120	100	120	153.33	152.5
130	120	110	140	135	146.67	135
110	117.5	130	115	132.5	170	150
120	125	127.5	110	142.5	161.67	132.5
120	135	135	140	147.5	153.33	137.5
140	120	150	147.5	170	157.5	135
95	102.5	110	100	107.5	188.33	125
120	145	115	110	150	175	135
160	140	165	180	172.5	155	112.5
130	150	155	150	167.5	150	100
150	135	115	150	175	168.75	115
142.5	145	152.5	140	172.5	131.25	111.67
137.5	130	135	140	140	176.67	127.5
147.5	150	147.5	145	162.5	153.33	122.5
160	135	155	155	148.33	158.75	130
135	132.5	130	120	135	147.5	145
147.5	145	135	132.5	150	145	130
130	122.5	122.5	120	130	140	107.5
140	142.5	140	147.5	150	135	102.5
147.5	145	135	132.5	150	145	130

contactnor15	contactnor20	contactnor25	contactnor30	contactnor35	contactnor40	contactnor45
132.5	130	122.5	120	115	115	115
112.5	110	102.5	102.5	100	90	95
130	112.5	120	110	100	105	110
137.5	130	122.5	112.5	110	110	105
135	125	117.5	120	117.5	110	110
130	132.5	125	120	110	110	110
125	125	115	115	115	112.5	125
135	120	115	120	115	110	115
130	125	120	110	120	110	110
120	112.5	105	107.5	110	105	110
122.5	120	125	112.5	115	110	105
100	95	85	75	90	85	75
97.5	100	85	80	90	80	102.5
140	130	115	90	110	105	100
107.5	80	80	82.5	75	80	75
112.5	110	115	107.5	102.5	110	102.5
115	110	100	105	100	100	102.5
127.5	110	107.5	120	102.5	105	100
120	107.5	100	92.5	100	95	97.5
112.5	112.5	110	135	110	107.5	105
105	97.5	92.5	90	95	90	92.5
97.5	90	87.5	85	82.5	80	82.5
112.5	112.5	110	135	110	107.5	105

contactnor50	contactnor55	contactnor60	contactnor65	contactnor70	contactnor75	contactnor80
120	115	120	115	115	117.5	115
90	95	95	135	100	90	90
100	95	100	105	95	95	100
100	97.5	100	100	105	95	95
112.5	115	110	110	110	115	115
110	110	107.5	110	115	105	110
115	110	120	112.5	110	112.5	115
107.5	110	100	110	105	105	110
110	100	105	105	105	105	115
105	100	102.5	110	115	110	115
105	110	105	105	110	105	102.5
95	85	75	77.5	95	77.5	75
105	90	85	87.5	85	85	85
115	105	110	92.5	100	100	95
85	77.5	90	90	75	70	77.5
105	95	105	102.5	105	107.5	107.5
90	100	105	97.5	95	100	97.5
100	100	105	110	105	100	100
95	95	97.5	100	95	82.5	100
92.5	125	110	105	112.5	105	92.5
92.5	92.5	92.5	95	92.5	95	95
82.5	85	80	80	77.5	75	77.5
92.5	125	110	105	112.5	105	92.5

contactnor85	contactnor90	contactnor95	contactnor10(	vel3kg5	vel3kg10	vel3kg15
115	115	115	117.5	5.93	6.71	7.38
90	95	95	105	5.48	7.07	8.03
100	95	90	102.5	5.4	6.62	7.37
95	97.5	100	102.5	5.19	6.79	7.75
112.5	110	110	115	5.48	6.65	7.23
112.5	100	115	127.5	5.29	6.54	7.34
115	117.5	115	120	5.45	6.52	7.16
107.5	100	115	110	5.07	6.69	7.26
110	115	110	80	5.6	6.73	7.65
107.5	105	110	122.5	5.75	6.96	7.66
105	105	110	115	5.09	6.54	7.58
90	77.5	80	82.5	4.25	6.38	7.43
90	100	90	95	3.74	5.72	6.68
95	100	100	105	4.3	6.18	7.06
80	72.5	95	97.5	4.41	6.1	6.98
110	107.5	110	110	4.16	5.78	6.62
100	97.5	100	102.5	3.76	5.7	6.53
105	100	100	98.33	3.62	6.58	6.8
92.5	97.5	90	100	4.31	5.91	6.73
105	115	115	120	3.94	5.44	6.45
95	97.5	100	102.5	3.79	5.62	6.71
77.5	80	82.5	90	4.15	6.85	7.37
105	115	115	120	3.94	5.44	6.45

vel3kg20	vel3kg25	vel3kg30	vel3kg35	vel3kg40	vel3kg45	vel3kg50
7.78	8.13	8.43	8.66	8.85	9	9.11
8.63	9.12	9.53	9.84	10.09	10.27	10.4
7.85	8.25	8.58	8.85	9.13	9.29	9.37
8.33	8.81	9.21	9.53	9.77	9.95	10.08
7.72	8.27	8.6	8.79	9.02	9.2	9.34
7.85	8.3	8.68	9	9.27	9.42	9.59
7.88	8.3	8.63	8.9	9.09	9.25	9.32
7.73	8.26	8.59	8.86	9.09	9.2	9.37
8.23	8.7	9.1	9.32	9.6	9.81	9.91
8.43	8.89	9.28	9.59	9.83	10.01	10.1
8.19	8.66	9.06	9.35	9.59	9.75	9.87
7.97	8.37	8.68	8.93	9.13	9.27	9.39
7.42	7.99	8.32	8.68	8.88	8.99	9.15
7.54	7.89	8.16	8.37	8.6	8.67	8.72
7.66	8.13	8.44	8.61	8.74	8.84	8.92
7.22	7.57	7.84	8.11	8.25	8.36	8.44
7.11	7.46	7.72	7.94	8.1	8.23	8.32
7.03	7.5	7.94	8.3	8.57	0.17	0.20
7.29	7.7	7.94	8.11	8.3	8.41	8.49
7.19	7.69	7.94	8.1	8.21	8.3	8.39
7.28	7.58	7.77	7.92	7.99	8.04	8.07
7.66	7.92	8.14	8.38	8.62	8.83	8.99
7.19	7.69	7.94	8.1	8.21	8.3	8.39

vel3kg55	vel3kg60	vel3kg65	vel3kg70	vel3kg75	vel3kg80	vel3kg85
9.17	9.2	9.23	9.23	9.21	9.16	9.1
10.47	10.53	10.56	10.57	10.57	10.57	10.57
9.46	9.52	9.54	9.55	9.55	9.51	9.47
10.15	10.21	10.26	10.28	10.28	10.31	10.33
9.44	9.5	9.53	9.54	9.54	9.51	9.5
9.73	9.79	9.85	9.89	9.89	9.86	9.82
9.39	9.43	9.45	9.46	9.46	9.45	9.45
9.43	9.49	9.52	9.52	9.52	9.46	9.43
10	10.06	10.09	10.08	10.05	10	10.00
10.2	10.25	10.26	10.25	10.25	10.18	10.11
9.92	9.96	9.97	9.96	9.95	9.89	9.85
9.44	9.5	9.53	9.55	9.55	9.56	9.56
9.22	9.29	9.33	9.4	9.45	9.48	9.48
8.77	8.81	8.83	8.84	8.83	8.82	8.8
9	9.04	9.07	9.07	9.05	9	8.89
8.5	8.54	8.58	8.6	8.62	8.63	8.61
8.37	8.4	8.42	8.42	8.42	8.42	8.42
0.20	0.20	0.25	0.25	0.20	0.20	0.20
8.55	8.59	8.63	8.65	8.66	8.66	8.64
8.45	8.51	8.56	8.58	8.58	8.55	8.46
8.11	8.18	8.22	8.25	8.24	8.19	8.08
9.05	8.99	8.9	8.76	8.64	8.65	8.77
8.45	8.51	8.56	8.58	8.58	8.55	8.46

vel3kg90	vel3kg95	vel3kg100	freq3kg5	freq3kg10	freq3kg15	freq3kg20
9.04	8.94	8.76	3.96	3.88	3.93	4.35
10.59	10.61	10.65	4.29	4.83	4.79	5.13
9.42	9.38	9.33	4	4.55	4.49	4.35
10.36	10.4	10.47	4.37	4.21	4.85	4.65
9.46	9.41	9.38	4.20	4.21	4.55	4.26
9.78	9.7	9.57	4.26	4.26	4.49	4.26
9.45	9.47	9.52	4.15	4.26	4.26	4.05
9.36	9.29	9.22	4.14	4.35	4.17	4.49
9.79	9.96	9.55	3.85	4	4	4.26
10.03	9.97	9.88	4.01	4.55	4.55	4.57
9.8	9.78	9.76	3.29	3.96	4.21	4.55
9.52	9.43	9.19	4.31	3.94	4.02	4.09
9.43	9.34	9.07	4.39	4.35	4.46	4.51
8.76	8.68	8.51	3.36	4.01	3.98	4.28
8.73	8.48	8	3.86	4.52	4.53	4.49
8.58	8.51	8.34	3.21	4.01	4.18	4.23
8.41	8.37	8.17	2.93	3.81	3.94	3.93
0.20	0.20	0.20	3.51	3.92	3.92	3.96
8.59	8.5	8.25	3.51	3.93	4.3	4.38
8.34	8.21	8.15	3.47	3.99	4.04	4.18
7.9	7.76	7.74	3.64	4.11	4.49	4.63
9	9.36	9.91	4.30	4.55	4.55	4.60
8.34	8.21	8.15	3.47	3.99	4.04	4.18

freq3kg25	freq3kg30	freq3kg35	freq3kg40	freq3kg45	freq3kg50	freq3kg55
3.89	4.17	4.21	4.17	4	4.17	4.26
4.85	4.35	4.85	5.13	4.71	4.65	5
4.55	4.65	4.6	4.65	4.65	4.76	4.6
4.65	4.44	4.76	4.65	4.71	4.76	4.88
4.35	4.08	4.65	4.35	4.17	4.35	4.76
4.35	4.55	4.4	4.08	4.55	4.35	4.35
4.26	4.26	4.44	4.3	4.26	4.35	4.26
4.4	4.44	4.3	4.35	4.65	4.55	4.26
4.21	4	4.35	4.08	4.17	4.08	4.26
4.55	4.55	4.76	4.65	4.44	4.65	4.71
4.45	4.35	4.5	4.88	4.55	4.26	4.88
4.16	4.3	4.22	4.32	4.24	4.24	4.34
4.49	4.29	4.13	4.53	4.48	4.48	4.44
4.13	3.96	4.37	4.15	4.32	4.34	4.25
4.7	4.67	4.53	4.69	4.75	4.65	4.59
4.2	4.31	4.28	4.42	4.29	4.16	4.34
3.95	4.04	4.14	4.1	4.15	4.16	4.15
4.04	4.12	4.35	4.00	4.00	4.08	4.08
4.55	4.61	4.62	4.52	4.33	4.49	4.64
4.21	4.22	4.42	3.91	4.29	4.29	4.25
4.46	4.6	4.69	4.69	4.49	4.86	4.48
4.55	4.76	4.55	4.65	4.60	4.60	4.65
4.21	4.22	4.42	3.91	4.29	4.29	4.25

freq3kg60	freq3kg65	freq3kg70	freq3kg75	freq3kg80	freq3kg85	freq3kg90
4.17	4.17	4.26	4.08	4.21	4.17	4.17
4.89	4.55	5	5	4.44	5.13	4.83
4.65	4.71	4.65	4.65	4.26	4.77	4.44
4.76	4.76	4.76	4.76	4.55	4.71	4.76
4.49	4.17	4.65	4.65	4.08	4.76	4.35
4.26	4.49	4.26	4.26	4.4	4.44	4.35
4.35	4.17	4.21	4.21	4.17	4.26	4.08
4.36	4.35	4.26	4.26	4.44	4.17	4.31
4.21	4.17	4.08	4.17	4.17	4.26	4.17
4.65	4.65	4.65	4.65	4.76	4.55	4.65
4.55	4.44	4.65	4.44	4.17	4.5	4.44
4.3	4.16	4.25	4.25	4.35	4.35	4.13
4.75	4.16	4.25	4.45	4.54	4.41	4.02
4.27	4.44	4.33	4	4.26	4.17	4.27
4.64	4.71	4.26	4.82	4.46	4.68	4.39
4.25	4.3	4.25	4.21	4.26	4.27	4.09
4.04	4.17	4.08	4	4.12	4.08	4
4.08	4.08	4.17	3.92	4.26	4.26	3.92
4.44	4.49	4.25	4.49	4.55	4.45	4.01
4.12	4.34	4	4.26	4.18	4.24	4.02
4.69	4.43	4.6	4.52	4.69	4.5	4.71
4.44	4.55	4.49	4.65	4.60	4.55	4.55
4.12	4.34	4	4.26	4.18	4.24	4.02

freq3kg95	freq3kg100	leng3kg5	leng3kg10	leng3kg15	leng3kg20	leng3kg25
3.74	3.51	1.5	1.73	1.88	1.79	2.09
4.65	5.13	1.29	1.47	1.69	1.68	1.89
4.65	4.35	1.36	1.46	1.64	1.81	1.81
4.55	4.71	1.2	1.61	1.61	1.79	1.89
3.64	4.26	1.34	1.59	1.67	1.78	1.92
4.35	4.13	1.24	1.53	1.63	1.84	1.91
4.08	3.81	1.32	1.53	1.68	1.95	1.95
4.35	4.04	1.23	1.54	1.74	1.72	1.88
4.26	4.08	1.46	1.68	1.91	1.94	2.07
4.55	4.04	1.43	1.53	1.68	1.85	1.96
4.44	4	1.54	1.65	1.8	1.8	1.95
3.95	3.87	1.07	1.62	1.85	1.95	2.01
4.79	4.05	0.85	1.32	1.5	1.65	1.78
3.79	3.82	1.27	1.54	1.77	1.76	1.92
4.4	4.3	1.14	1.35	1.56	1.72	1.65
4.27	3.96	1.34	1.44	1.59	1.71	1.8
3.86	3.94	1.29	1.5	1.66	1.81	1.89
3.92	4.08	0.91	1.94	1.75	1.90	1.86
4.12	4.35	1.23	1.5	1.57	1.67	1.69
4.11	3.71	1.13	1.37	1.6	1.72	1.83
4.58	2.69	1.04	1.37	1.49	1.57	1.7
4.35	4.17	1.03	1.73	1.66	1.72	1.76
4.11	3.71	1.13	1.37	1.6	1.72	1.83

leng3kg30	leng3kg35	leng3kg40	leng3kg45	leng3kg50	leng3kg55	leng3kg60
2.02	2.06	2.12	2.25	2.19	2.15	2.21
2.19	2.04	1.97	2.18	2.24	2.09	2.16
1.85	1.93	1.96	2	1.97	2.06	2.05
2.07	2	2.1	2.11	2.12	2.08	2.15
2.10	2.02	2.02	2.18	2.07	2.09	2.19
1.91	2.05	2.27	2.07	2.21	2.24	2.3
2.03	2	2.11	2.17	2.14	2.21	2.17
1.93	2.06	2.09	1.98	2.06	2.22	2.18
2.28	2.14	2.35	2.35	2.43	2.35	2.39
2.04	2.01	2.11	2.25	2.17	2.17	2.2
2.08	2.08	1.97	2.15	2.32	2.03	2.19
2.02	2.11	2.11	2.19	2.21	2.18	2.21
1.94	2.1	1.97	2.01	2.04	2.08	1.96
2.06	1.92	2.07	2.01	2.01	2.07	2.07
1.81	1.9	1.87	1.86	1.92	1.96	1.95
1.82	1.9	1.87	1.95	2.03	1.96	2.01
1.91	1.92	1.98	1.98	2	2.01	2.08
1.97	1.91	2.21	0.04	0.05	0.05	0.05
1.72	1.76	1.83	1.94	1.89	1.84	1.94
1.88	1.83	2.1	1.94	1.95	1.99	2.07
1.69	1.69	1.7	1.79	1.66	1.81	1.74
1.73	1.86	1.87	1.90	1.96	1.92	2.07
1.88	1.83	2.1	1.94	1.95	1.99	2.07

leng3kg65	leng3kg70	leng3kg75	leng3kg80	leng3kg85	leng3kg90	leng3kg95
2.21	2.17	2.26	2.18	2.18	2.17	2.39
2.32	2.11	2.11	2.38	2.06	2.2	2.28
2.03	2.05	2.05	2.24	1.99	2.12	2.02
2.16	2.16	2.16	2.27	2.2	2.18	2.29
2.19	2.01	2.19	2.29	2.17	2.05	2.34
2.19	2.32	2.32	2.24	2.21	2.25	2.23
2.27	2.25	2.25	2.27	2.22	2.32	2.32
2.19	2.24	2.24	2.13	2.26	2.18	2.14
2.42	2.47	2.41	2.4	2.35	2.35	2.34
2.21	2.2	2.2	2.14	2.22	2.16	2.19
2.24	2.14	2.24	2.37	2.19	2.21	2.2
2.29	2.25	2.25	2.2	2.2	2.3	2.39
2.24	2.21	2.13	2.09	2.15	2.35	1.95
1.99	2.05	2.21	2.07	2.11	2.05	2.29
1.93	2.13	1.88	2.02	1.9	1.99	1.93
2	2.02	2.05	2.03	2.02	2.1	1.99
2.02	2.06	2.1	2.04	2.06	2.1	2.17
0.06	0.06	0.05	0.05	0.05	0.05	0.05
1.92	2.03	1.93	1.9	1.94	2.14	2.07
1.97	2.15	2.01	2.05	2	2.07	2
1.86	1.8	1.83	1.75	1.8	1.68	1.69
1.96	1.97	1.88	1.86	1.93	1.96	2.13
1.97	2.15	2.01	2.05	2	2.07	2

leng3kg100	flight3kg5	flight3kg10	flight3kg15	flight3kg20	flight3kg25	flight3kg30
2.5	87.5	105	125	110	122.5	115
2.08	80	87.5	95	90	105	130
2.15	75	95	107.5	115	115	120
2.23	63.33	92.5	77.5	85	90	105
2.35	81.67	97.5	95	110	107.5	130
2.32	61.67	77.5	85	100	107.5	105
2.5	70	80	85	105	115	107.5
2.28	91.67	102.5	115	112.5	115	110
2.34	105	105	122.5	125	122.5	155
2.45	71.67	80	90	100	100	107.5
2.44	140	117.5	107.5	105	105	115
2.37	83.33	117.5	125	140	160	155
2.26	115	117.5	122.5	127.5	127.5	125
2.23	100	115	122.5	115	120	135
1.87	128.67	85	117.5	125	117.5	140
2.11	140.67	105	115	125	130	127.5
2.13	186.67	120	135	142.5	150	140
0.05	115.25	125	127.5	142.5	137.5	137.5
1.92	125	122.5	115	120	120	110
2.22	120	120	137.5	142.5	135	120
1.89	102	98.33	112.5	112.5	110	122.5
2.30	97.5	117.5	122.5	127.5	132.5	125
2.22	120	120	137.5	142.5	135	120

flight3kg35	flight3kg40	flight3kg45	flight3kg50	flight3kg55	flight3kg60	flight3kg65
105	110	125	125	115	120	120
115	100	115	125	110	115	130
115	117.5	115	115	117.5	115	117.5
102.5	110	107.5	110	105	107.5	115
100	117.5	130	122.5	105	117.5	125
110	130	110	112.5	120	120	117.5
115	117.5	110	120	122.5	115	125
120	125	120	110	125	120	120
145	145	130	150	135	142.5	135
110	112.5	120	115	112.5	115	115
120	115	122.5	135	115	122.5	130
150	140	152.5	165	145	157.5	165
130	127.5	130	132.5	130	140	135
122.5	130	120	127.5	140	135	130
145	142.5	135	147.5	145	140	142.5
130	125	135	145	132.5	135	135
135	147.5	145	142.5	145	152.5	145
125	152.5	155	142.5	142.5	142.5	140
117.5	125	140	132.5	110	135	130
127.5	155	137.5	140	155	147.5	135
117.5	122.5	120	135	122.5	135	115
137.5	135	135	135	130	145	140
127.5	155	137.5	140	155	147.5	135

flight3kg70	flight3kg75	flight3kg80	flight3kg85	flight3kg90	flight3kg95	flight3kg100
115	120	117.5	115	115	150	150
115	115	135	105	115	125	100
120	120	135	117.5	130	120	130
110	110	120	115	110	120	110
100	100	135	105	120	165	120
130	130	120	115	115	120	122.5
120	120	130	122.5	125	135	140
125	125	120	130	122.5	115	147.5
140	140	135	135	135	130	140
115	115	115	117.5	115	120	140
120	130	135	125	125	120	150
150	150	155	140	157.5	175	177.5
140	145	140	142.5	130	175	147.5
135	140	140	145	142.5	165	175
140	135	150	140	155	150	155
135	140	135	132.5	145	132.5	152.5
147.5	155	150	150	160	160	167.5
135	150	135	135	155	155	135
145	132.5	130	137.5	140	142.5	140
155	142.5	150	142.5	160	152.5	165
127.5	127.5	130	127.5	130	120	135
145	140	140	142.5	140	147.5	150
155	142.5	150	142.5	160	152.5	165

contact3kg5	contact3kg10	contact3kg15	contact3kg20	contact3kg25	contact3kg30	contact3kg35
165	152.5	130	120	135	125	132.5
158.33	120	115	105	102.5	100	92.5
178.33	125	115	115	105	95	102.5
168.33	145	130	130	125	120	107.5
156.67	140	125	125	122.5	115	115
173.33	157.5	137.5	135	122.5	115	117.5
171.67	155	150	142.5	120	127.5	110
150	127.5	125	110	112.5	115	112.5
155	145	127.5	110	115	95	85
178.33	140	130	120	120	112.5	100
165	135	130	115	120	115	102.5
145	125	117.5	100	77.5	75	85
140	102.5	97.5	92.5	92.5	85	80
170	125	122.5	115	120	115	105
146.67	127.5	100	95	82.5	72.5	75
176.67	135	120	107.5	105	102.5	102.5
165	132.5	112.5	107.5	100	105	105
170	130	127.5	110	110	105	105
158.33	122.5	112.5	105	97.5	105	97.5
150	120	102.5	92.5	100	115	97.5
141.67	120	102.5	97.5	100	92.5	92.5
135	102.5	97.5	90	87.5	85	82.5
150	120	102.5	92.5	100	115	97.5

contact3kg40	contact3kg45	contact3kg50	contact3kg55	contact3kg60	contact3kg65	contact3kg70
130	125	115	120	120	120	120
95	97.5	90	90	90	90	85
97.5	100	95	100	100	95	95
105	105	100	100	102.5	95	100
112.5	110	107.5	105	105	115	115
115	110	117.5	110	115	105	105
115	125	110	112.5	115	115	117.5
105	95	110	110	110	110	110
100	110	95	100	95	105	105
102.5	105	100	100	100	100	100
90	97.5	100	90	97.5	95	95
90	82.5	70	85	75	75	85
95	92.5	92.5	92.5	100	80	75
110	120	102.5	95	100	95	97.5
70	75	67.5	72.5	75	70	95
100	97.5	95	97.5	100	97.5	100
95	95	97.5	95	95	95	97.5
97.5	95	102.5	102.5	102.5	105	105
95	90	90	105	90	92.5	90
100	95	92.5	80	95	95	95
92.5	95	85	90	90	95	90
80	82.5	82.5	85	80	80	77.5
100	95	92.5	80	95	95	95

contact3kg75	contact3kg80	contact3kg85	contact3kg90	contact3kg95	contact3kg10(	vel4kg5
125	120	125	125	117.5	135	5.24
85	90	90	92.5	90	95	5.69
95	100	92.5	95	95	100	5.36
100	100	97.5	100	100	102.5	5.19
115	110	105	110	110	115	
105	107.5	110	115	110	120	5.48
117.5	110	112.5	120	110	122.5	5.52
110	105	110	110	115	100	4.87
100	105	100	105	105	105	5.52
100	95	102.5	100	100	107.5	5.94
95	105	97.5	100	105	100	5.54
85	75	90	85	80	85	3.93
82.5	100	90	80	95	92.5	4.15
110	95	95	92.5	100	90	3.92
72.5	75	75	75	80	80	4.54
97.5	100	102.5	100	102.5	102.5	4.1
95	92.5	95	90	100	100	3.89
105	100	100	100	100	110	4.39
90	90	87.5	110	102.5	95	4.37
92.5	90	95	90	92.5	105	4.27
95	90	95	90	100	90	3.81
75	77.5	77.5	80	82.5	90	3.99
92.5	90	95	90	92.5	105	4.39

vel4kg10	vel4kg15	vel4kg20	vel4kg25	vel4kg30	vel4kg35	vel4kg40
6.4	7.26	7.81	8.25	8.61	8.89	9.11
7.07	7.99	8.58	9.09	9.53	9.89	10.2
6.66	7.47	7.98	8.4	8.74	9.02	9.31
6.79	7.75	8.38	8.9	9.3	9.65	9.89
6.72	7.36	7.9	8.52	8.92	9.25	9.52
6.58	7.24	7.95	8.4	8.76	9.15	9.23
6.58	7.19	7.86	8.29	8.65	8.95	9.19
6.74	7.69	8.27	8.72	9.09	9.29	9.51
6.9	7.72	8.41	8.87	9.26	9.6	9.87
6.98	7.45	8.22	8.7	9.07	9.38	9.62
6.27	7.4	8.01	8.45	8.86	9.12	9.36
5.82	6.81	7.58	8.16	8.49	8.75	8.96
5.97	7.05	7.62	8.03	8.43	8.65	8.8
6.5	7.45	8.13	8.51	8.78	8.98	9.13
5.95	6.79	7.39	7.74	8.01	8.24	8.41
5.76	6.63	7.25	7.61	7.88	8.11	8.27
6.14	6.95	7.52	7.73	7.98	8.16	8.31
6	6.8	7.4	7.85	8.17	8.36	8.52
5.9	6.79	7.41	7.74	7.98	8.18	8.33
5.97	6.76	7.32	7.73	7.94	8.12	8.24
6.69	7.21	7.5	7.69	7.9	8.22	8.46
6.14	6.95	7.52	7.73	7.98	8.16	8.31

vel4kg45	vel4kg50	vel4kg55	vel4kg60	vel4kg65	vel4kg70	vel4kg75
9.23	9.35	9.45	9.48	9.5	9.51	9.49
10.37	10.58	10.75	10.82	10.9	10.94	10.95
9.47	9.55	9.63	9.69	9.72	9.72	9.72
10.07	10.2	10.27	10.33	10.38	10.4	10.4
9.67	9.85	9.99	10.05	10.11	10.13	10.12
9.42	9.57	9.63	9.7	9.72	9.71	9.67
9.38	9.47	9.58	9.65	9.67	9.68	9.67
9.68	9.76	9.82	9.87	9.9	9.9	9.91
10.12	10.25	10.4	10.5	10.56	10.57	10.55
9.81	9.93	10.01	10.04	10.04	10.01	9.97
9.49	9.59	9.68	9.76	9.79	9.85	9.87
9.11	9.24	9.35	9.46	9.52	9.61	9.68
8.91	8.98	9.03	9.05	9.07	9.09	9.1
9.25	9.32	9.36	9.4	9.43	9.45	9.45
8.55	8.65	8.72	8.76	8.79	8.81	8.82
8.4	8.49	8.54	8.57	8.59	8.61	8.63
8.4	8.46	8.48	8.5	8.51	8.51	8.52
8.63	8.7	8.78	8.83	8.87	8.9	8.94
8.46	8.56	8.66	8.71	8.72	8.71	8.67
8.32	8.4	8.45	8.47	8.49	8.49	8.49
8.65	8.81	8.84	8.78	8.67	8.53	8.41
8.4	8.46	8.48	8.5	8.51	8.51	8.52

vel4kg80	vel4kg85	vel4kg90	vel4kg95	vel4kg100	freq4kg5	freq4kg10
9.47	9.44	9.42	9.39	9.38	3.52	3.92
10.94	10.9	10.85	10.77	10.63	4.08	4.65
9.7	9.69	9.67	9.66	9.66	4.12	4.6
10.43	10.45	10.48	10.52	10.59	4.33	4.45
10.09	10.03	9.93	9.85	9.7	4.34	4.26
9.6	9.54	9.47	9.35	9.17	3.98	4.35
9.63	9.45	9.57	9.53	9.38	3.97	4
9.91	9.92	9.94	9.97	10.05	4	3.96
10.48	10.36	10.26	10.05	9.71	4.79	4.36
9.9	9.8	9.72	9.6	9.46	4.35	4.04
9.88	9.9	9.89	9.85	9.69	3.29	3.85
9.71	9.7	9.65	9.48	9.21	5.45	4.37
9.1	9.1	9.07	9.01	8.83	3.55	3.62
9.43	9.38	9.28	9.11	8.72	4	4.37
8.83	8.84	8.84	8.81	8.7	3.2	3.97
8.64	8.66	8.64	8.55	8.34	3.11	3.84
8.52	8.52	8.47	8.32	8.03	3.46	3.73
8.96	8.97	8.94	8.87	8.67	3.52	4.13
8.6	8.52	8.47	8.54	8.98	3.65	3.89
8.46	8.43	8.36	8.25	8.11	3.99	4.26
8.37	8.49	8.72	9.08	9.63	3.66	4.43
8.52	8.52	8.47	8.32	8.03	3.46	3.73

freq4kg15	freq4kg20	freq4kg25	freq4kg30	freq4kg35	freq4kg40	freq4kg45
4.1	4	4.12	4.17	4.17	4.17	4.08
4.65	4.65	4.82	4.35	4.76	5.13	4.65
4.55	4.44	4.65	4.65	4.65	4.76	4.44
4.67	4.76	4.6	4.6	4.88	4.77	4.44
4.44	4.45	4.5	4.44	4.4	4.44	4.55
4.15	4.21	4.17	4.22	4.31	4.08	4.31
4.35	4.4	4.26	4.35	4.44	4.31	4.35
4.13	4.08	4.17	4	4.17	4.12	4.08
4.55	4.71	4.55	4.72	5	4.83	4.65
4.30	4.55	4.65	4.49	4.55	4.55	4.40
3.98	4.26	4.07	5.83	3.8	3.82	4.06
4.54	4.57	4.4	4.21	4.37	4.63	4.42
3.97	4.17	4.2	4.18	4.14	4.33	4.34
4.59	4.58	4.71	4.85	4.52	4.8	4.75
4.16	4.23	4.2	4.36	4.41	4.28	4.24
3.98	4.05	4.11	4.08	4.05	4.1	4.24
3.07	3.72	3.96	4.22	3.86	3.98	4
4.39	4.42	4.6	4.67	4.52	4.63	4.34
4.02	4.07	4.21	4.22	4.23	4.23	4.33
4.68	4.74	4.6	4.78	4.65	3.97	4.83
4.5	4.56	4.61	4.56	4.5	4.61	4.57
3.07	3.72	3.96	4.22	3.86	3.98	4

freq4kg50	freq4kg55	freq4kg60	freq4kg65	freq4kg70	freq4kg75	freq4kg80
4.12	4.17	4.26	4.26	4.17	4.26	4.12
4.55	5.00	4.76	4.44	4.88	4.88	4.40
4.88	4.71	4.65	4.71	4.76	4.71	4.55
4.76	4.77	4.44	4.88	4.88	4.55	4.71
4.46	4.44	4.35	4.44	4.17	4.44	4.26
4.44	4.08	4.18	4.35	4.17	4.21	4.26
4.65	4.41	4.08	4.65	4.44	4	4.36
4.08	4.17	4.09	4.08	4.26	4.08	4.12
5	4.77	4.65	4.76	4.76	4.65	4.82
4.35	4.65	4.49	4.40	4.44	4.65	4.21
4.33	4.29	4.44	4.08	4	4.54	3.92
4.33	4.29	4.53	4.43	4.38	4.44	4.4
4.29	4.25	4.54	4.26	4.35	4.3	4.35
5.12	4.49	4.65	4.7	4.88	4.66	5
4.29	4.34	4.34	4.16	4.25	4.25	4.26
4.17	3.92	4.08	4.08	4.16	4.08	4.16
4.16	3.84	4.2	4.41	4	4	4.09
4.44	4.64	4.49	4.44	4.54	4.54	4.44
4.24	4.34	4.25	4.17	4.31	4.2	4.27
4.72	4.69	4.69	4.74	4.66	4.63	4.75
4.59	4.66	4.46	4.57	4.52	4.66	4.59
4.16	3.84	4.2	4.41	4	4	4.09

freq4kg85	freq4kg90	freq4kg95	freq4kg100	leng4kg5	leng4kg10	leng4kg15
4.17	4.26	3.96	3.51	1.49	1.63	1.78
5.13	4.88	4.55	5.26	1.39	1.52	1.72
4.67	4.65	4.65	4.31	1.31	1.45	1.64
4.88	4.44	4.88	4.66	1.20	1.53	1.67
4.45	4.35	4.26	4.31	1.26	1.58	1.66
4.08	4.26	4.04	3.86	1.39	1.51	1.75
4.26	4.26	4.26	4.17	1.22	1.64	1.65
4	3.92	4.08	3.93	1.38	1.7	1.87
4.76	4.55	4.71	4.4	1.27	1.59	1.7
4.35	4.65	4.44	3.85	1.27	1.73	1.73
5	4.06	4.89	4.4	1.18	1.63	1.86
4.26	4.37	4.34	3.91	0.85	1.33	1.52
4.2	4.01	4.51	3.92	1.12	1.65	1.78
4.62	5.03	4.75	4.06	0.97	1.49	1.62
4.17	4.3	4.18	4.12	1.3	1.5	1.63
4.04	3.93	3.99	3.7	1.26	1.5	1.66
3.93	3.94	4.22	3.94	1.26	1.65	2.35
4.26	4.42	4.37	4.27	1.26	1.48	1.55
4.18	4.16	4.14	3.5	1.17	1.52	1.69
5.43	4.75	3.82	3.94	0.97	1.43	1.53
4.51	4.49	4.3	4.03	1.04	1.51	1.61
3.93	3.94	4.22	3.94	1.26	1.65	2.35

leng4kg20	leng4kg25	leng4kg30	leng4kg35	leng4kg40	leng4kg45	leng4kg50
1.95	2	2.07	2.13	2.19	2.26	2.27
1.84	1.89	2.19	2.08	1.99	2.23	2.33
1.8	1.81	1.88	1.94	1.95	2.13	1.96
1.76	1.94	2.02	1.98	2.08	2.27	2.14
1.78	1.9	2.01	2.1	2.14	2.13	2.22
1.89	2.02	2.08	2.13	2.26	2.19	2.15
1.79	1.95	1.99	2.01	2.14	2.16	2.04
2.03	2.09	2.27	2.23	2.31	2.37	2.39
1.79	1.95	1.97	1.92	2.05	2.18	2.05
1.81	1.87	2.02	2.06	2.12	2.23	2.28
1.88	2.13	1.52	2.46	2.45	2.34	2.21
1.7	1.86	2.02	2.01	1.94	2.06	2.13
1.83	1.91	2.02	2.09	2.03	2.05	2.09
1.78	1.81	1.81	1.99	1.9	1.95	1.82
1.75	1.84	1.84	1.87	1.97	2.02	2.02
1.79	1.85	1.93	2	2.02	1.98	2.04
0.99	1.95	1.89	2.11	2.09	2.11	2.03
1.68	1.71	1.75	1.85	1.84	1.99	1.92
1.82	1.84	1.89	1.93	1.97	1.95	1.61
1.61	1.74	1.37	1.78	2.07	1.78	1.83
1.64	1.67	1.74	1.83	1.83	1.89	1.92
0.99	1.95	1.89	2.11	2.09	2.11	2.03

leng4kg55	leng4kg60	leng4kg65	leng4kg70	leng4kg75	leng4kg80	leng4kg85
2.27	2.23	2.23	2.28	2.23	2.3	2.27
2.15	2.27	2.45	2.24	2.24	2.49	2.13
2.05	2.08	2.07	2.04	2.07	2.14	2.08
2.16	2.32	2.13	2.13	2.29	2.22	2.14
2.25	2.31	2.28	2.43	2.28	2.37	2.26
2.36	2.33	2.24	2.33	2.3	2.26	2.34
2.18	2.36	2.08	2.2	2.42	2.21	2.22
2.36	2.42	2.43	2.33	2.43	2.4	2.48
2.18	2.26	2.22	2.22	2.27	2.17	2.18
2.15	2.23	2.28	2.25	2.14	2.35	2.25
2.26	2.2	2.4	2.46	2.17	2.52	1.98
2.18	2.09	2.15	2.19	2.18	2.21	2.28
2.13	1.99	2.13	2.09	2.12	2.09	2.14
2.09	1.88	2.01	1.94	2.03	1.88	2.03
2.01	2.02	2.11	2.07	2.07	2.08	2.12
2.18	2.1	2.11	2.07	2.11	2.08	2.14
2.21	2.18	2.23	2.13	2.13	2.08	2.17
1.89	1.97	2	1.96	1.97	2.02	2.11
2	2.05	2.09	2.26	1.97	2.01	2.04
1.86	1.53	1.85	2.25	1.89	1.83	1.55
1.9	1.97	1.9	1.89	1.8	1.82	1.88
2.21	2.18	2.23	2.13	2.13	2.08	2.17

leng4kg90	leng4kg95	leng4kg100	flight4kg5	flight4kg10	flight4kg15	flight4kg20
2.21	2.37	2.67	62.5	80	97.5	115
2.22	2.37	2.02	80	87.5	95	90
2.08	2.08	2.25	90	100	105	115
2.36	2.16	2.28	68.33	85	82.5	90
2.28	2.32	2.26	66.67	90	90	90
2.23	2.31	2.38	86.67	95	117.5	115
2.25	2.24	2.25	76.67	115	110	107.5
2.53	2.44	2.56	80	107.5	122.5	130
2.26	2.14	2.21	55	75	90	92.5
2.09	2.16	2.46	90	117.5	107.5	105
2.47	2.01	1.84	93.33	120	137.5	135
2.21	2.19	2.35	136.75	115	110	120
2.26	2	2.25	120	130	122.5	130
1.85	1.92	2.18	109.75	97.5	112.5	115
2.05	2.11	2.12	159	107.5	127.5	127.5
2.2	2.15	2.26	168.33	130	137.5	135
2.15	1.98	1.98	135	140	152.5	145
2.03	2.03	2.03	108.33	105	127.5	120
2.03	2.07	2.6	113.33	122.5	137.5	142.5
1.85	2.16	2.1	104.67	112.5	115	125
1.94	2.12	2.36	96.25	117.5	122.5	127.5
2.15	1.98	1.98	135	140	152.5	145

flight4kg25	flight4kg30	flight4kg35	flight4kg40	flight4kg45	flight4kg50	flight4kg55
112.5	110	117.5	120	130	125	125
105	130	115	100	115	125	110
112.5	115	117.5	112.5	125	115	117.5
95	105	100	105	120	110	112.5
97.5	105	120	125	105	117.5	115
125	120	122.5	130	122.5	115	125
115	115	110	117.5	115	110	112.5
125	150	135	135	145	145	140
105	105	105	100	110	105	110
105	115	120	115	122.5	135	115
130	165	162.5	145	180	170	155
127.5	135	112.5	145	137.5	135	140
125	122.5	145	130	125	135	135
130	140	120	127.5	137.5	130	140
130	130	130	135	135	132.5	130
140	145	145	147.5	145	147.5	160
145	150	152.5	155	152.5	140	165
120	130	125	122.5	120	130	130
135	132.5	140	125	145	141.67	135
127.5	170	122.5	90	125	127.5	127.5
125	132.5	137.5	135	135	135	130
145	150	152.5	155	152.5	140	165

flight4kg60	flight4kg65	flight4kg70	flight4kg75	flight4kg80	flight4kg85	flight4kg90
120	117.5	120	115	122.5	125	120
115	130	115	115	135	105	115
115	112.5	115	117.5	125	115	125
120	110	110	120	117.5	110	125
115	112.5	130	115	130	120	120
122.5	125	130	122.5	120	130	120
115	105	115	125	120	125	120
145	135	135	140	140	145	155
115	107.5	110	115	112.5	110	110
122.5	130	120	130	135	125	125
155	145	155	165	150	145	142.5
125	140	150	150	135	145	145
110	137.5	140	130	140	140	130
130	147.5	150	130	140	147.5	115
130	140	137.5	135	140	140	135
150	145	145	150	140	155	160
147.5	140	150	155	135	150	160
130	130	130	130	132.5	125	135
142.5	145	135	132.5	150	162.5	140
175	127.5	85	135	132.5	165	137.5
145	140	145	140	140	142.5	140
147.5	140	150	155	135	150	160

flight4kg95	flight4kg100	contact4kg5	contact4kg10	contact4kg15	contact4kg20	contact4kg25
135	160	222.5	175	147.5	135	130
125	100	165	127.5	120	125	102.5
120	132.5	155	117.5	115	110	102.5
110	117.5	163.33	140	132.5	120	122.5
125	127.5	165	145	135	135	125
132.5	135	165	135	125	122.5	115
125	130	175	135	120	120	120
145	150	170	145	120	115	115
120	120	157.5	155	130	120	115
120	150	140	130	125	115	110
145	142.5	203.33	122.5	112.5	95	102.5
150	155	141.25	110	112.5	92.5	95
135	170	136.67	132.5	122.5	105	110
142.5	140	145	102.5	97.5	97.5	80
135	142.5	151.67	135	107.5	105	105
157.5	185	161.67	120	107.5	107.5	100
160	175	151.67	120	115	105	105
120	140	136.67	105	100	100	92.5
145	167.5	143.33	117.5	107.5	100	100
80	152.5	135	102.5	95	90	90
147.5	150	118.75	100	100	92.5	85
160	175	151.67	120	115	105	105

contact4kg30	contact4kg35	contact4kg40	contact4kg45	contact4kg50	contact4kg55	contact4kg60
130	122.5	120	115	117.5	115	115
100	95	95	100	95	90	95
100	97.5	97.5	100	90	95	100
112.5	105	105	105	100	97.5	105
120	107.5	100	115	107.5	110	115
117.5	110	115	110	110	120	117.5
115	115	115	115	105	115	130
100	105	107.5	100	100	100	100
107.5	95	107.5	105	95	100	100
107.5	100	105	105	95	100	100
95	87.5	80	80	75	72.5	85
80	90	85	87.5	90	90	100
115	95	100	105	97.5	100	110
70	95	87.5	75	70	75	75
97.5	95	97.5	100	100	100	100
97.5	100	95	90	92.5	95	95
85	105	95	97.5	100	95	105
85	90	100	90	92.5	90	95
100	100	105	90	91.67	95	92.5
90	90	85	87.5	87.5	90	90
87.5	100	90	92.5	87.5	105	90
85	105	95	97.5	100	95	105

contact4kg65	contact4kg70	contact4kg75	contact4kg80	contact4kg85	contact4kg90	contact4kg95
117.5	120	120	120	115	115	117.5
95	90	90	92.5	90	90	95
100	95	95	95	100	90	95
95	95	100	95	95	100	95
112.5	110	110	105	105	110	110
105	110	115	115	115	115	115
110	112.5	125	110	110	115	110
110	100	105	102.5	105	100	100
95	100	95	100	100	90	102.5
97.5	105	85	102.5	105	90	105
80	70	85	70	110	97.5	75
85	77.5	85	92.5	85	95	92.5
97.5	90	102.5	90	95	95	87.5
70	80	72.5	75	75	80	80
100	97.5	100	95	100	97.5	105
100	95	95	100	92.5	95	95
105	100	95	110	105	95	107.5
85	92.5	100	95	90	100	105
95	125	95	85	77.5	100	97.5
92.5	95	85	90	90	92.5	85
90	95	100	95	102.5	100	95
105	100	95	110	105	95	107.5

contact4kg10(	vel5kg5	vel5kg10	vel5kg15	vel5kg20	vel5kg25	vel5kg30
125	5.57	6.66	7.31	7.85	8.3	8.67
90	5.63	7.26	8.25	8.86	9.36	9.8
100	6.12	7.1	7.7	8.38	8.91	9.23
97.5	5.34	6.96	7.69	8.31	8.81	9.23
	5.8	6.79	7.32	7.79	8.34	8.69
105	5.51	6.87	7.54	8.12	8.76	9.15
125	5.09	6.63	7.45	8.31	8.8	9.04
110	5.01	6.64	7.26	7.92	8.36	8.73
105	5.52	6.81	7.86	8.47	8.81	9.22
112.5	5.79	7.02	7.75	8.57	9.08	9.48
110	5.71	7.15	7.62	8.39	8.87	9.24
110	4.15	6.33	7.46	8.27	8.73	9.08
95	4.2	6.33	7.31	7.82	8.36	8.68
87.5	3.82	6.01	7.1	7.84	8.24	8.53
80	4.54	6.5	7.45	8.13	8.51	8.78
102.5	4.34	5.97	6.89	7.39	7.78	8.17
90	3.98	5.88	6.74	7.36	7.74	8.03
95	4.42	6.45	7.18	7.69	8.1	8.4
102.5	4.37	6.31	7.12	7.71	8.05	8.29
102.5	3.98	5.71	6.69	7.44	7.85	8.19
92.5	4.19	6.4	7.13	7.59	7.9	8.08
90	4.13	6.83	7.35	7.64	7.83	8.04
95	4.42	6.45	7.18	7.69	8.1	8.4

vel5kg35	vel5kg40	vel5kg45	vel5kg50	vel5kg55	vel5kg60	vel5kg65
8.96	9.25	9.31	9.44	9.53	9.56	9.58
10.04	10.33	10.55	10.72	10.85	10.9	10.97
9.4	9.62	9.83	9.95	10	10.04	10.06
9.57	9.83	10.04	10.15	10.28	10.38	10.43
9.01	9.26	9.48	9.66	9.76	9.86	9.94
9.37	9.65	9.88	10	10.13	10.23	10.27
9.33	9.54	9.63	9.7	9.73	9.73	9.69
9.04	9.28	9.47	9.62	9.73	9.78	9.81
9.55	9.72	9.91	10.05	10.11	10.14	10.16
9.81	10	10.21	10.37	10.46	10.52	10.53
9.55	9.79	9.98	10.1	10.18	10.21	10.21
9.35	9.55	9.7	9.78	9.87	9.94	9.99
8.94	9.15	9.3	9.45	9.58	9.69	9.79
8.76	8.91	9.03	9.1	9.15	9.19	9.21
8.98	9.13	9.25	9.32	9.36	9.4	9.43
8.38	8.52	8.64	8.73	8.8	8.85	8.91
8.27	8.44	8.58	8.67	8.73	8.76	8.77
8.62	8.77	8.88	8.94	8.99	9.03	9.06
8.56	8.71	8.83	8.93	9	9.06	9.1
8.47	8.62	8.72	8.79	8.83	8.87	8.88
8.23	8.39	8.49	8.56	8.62	8.63	8.62
8.36	8.58	8.77	8.93	8.96	8.9	8.79
8.62	8.77	8.88	8.94	8.99	9.03	9.06

vel5kg70	vel5kg75	vel5kg80	vel5kg85	vel5kg90	vel5kg95	vel5kg100
9.57	9.55	9.51	9.45	9.41	9.37	9.3
11.01	11.03	11.05	11.07	11.09	11.13	11.19
10.05	10.03	9.99	9.96	9.88	9.8	9.73
10.49	10.55	10.58	10.63	10.7	10.76	10.87
9.96	9.96	9.92	9.84	9.72	9.63	9.43
10.31	10.32	10.32	10.31	10.3	10.28	10.26
9.65	9.63	9.6	9.6	9.68	9.68	9.83
9.83	9.81	9.77	9.71	9.62	10	9.98
10.15	10.13	10.1	10.07	10.03	10.17	10.04
10.53	10.49	10.43	10.37	10.27	10.17	10.04
10.18	10.14	10.07	9.97	9.89	9.77	9.63
10.03	10.1	10.15	10.16	10.12	9.98	9.58
9.87	9.92	9.96	9.96	9.92	9.78	9.5
9.23	9.25	9.27	9.29	9.29	9.25	9.13
9.45	9.45	9.43	9.38	9.28	9.11	8.72
8.96	8.99	9	8.99	8.94	8.77	8.56
8.78	8.79	8.8	8.81	8.8	8.76	8.58
9.08	9.1	9.11	9.09	9.05	8.88	8.53
9.14	9.16	9.17	9.16	9.12	9.02	8.82
8.91	8.93	8.93	8.91	8.83	8.68	8.35
8.58	8.54	8.47	8.42	8.36	8.31	8.22
8.65	8.53	8.49	8.63	8.86	9.22	9.77
9.08	9.1	9.11	9.09	9.05	8.88	8.53

freq5kg5	freq5kg10	freq5kg15	freq5kg20	freq5kg25	freq5kg30	freq5kg35
3.88	4	4.04	4.08	4.08	4.26	4.13
4.33	4.76	4.88	5.13	4.84	4.55	5
4.44	4.55	4.49	4.55	4.76	4.65	4.82
4	4.52	4.76	4.46	4.55	4.49	4.76
4.13	4.35	4.44	4.45	4.46	4.26	4.5
4.38	4.45	4.35	4.5	4.55	4.55	4.35
3.92	4.17	4.17	4.26	4.26	4.44	4.17
4.06	4.08	4.26	4.31	4.55	4.26	4.44
4.01	3.81	4.08	4	4.08	4.17	4.17
4.37	4.65	4.55	4.5	4.65	4.61	4.88
4.26	4.04	4.30	4.55	4.65	4.49	4.55
3.28	5.83	4.2	4.37	4.18	4.34	4.77
3.66	4.65	4.52	4.61	4.59	4.3	4.32
3.43	3.93	4.14	4.32	4.39	4.27	4.42
4	4.37	4.59	4.58	4.71	4.85	4.52
3.33	3.84	4.19	4.09	4.2	4.18	4.32
3.12	3.96	4.06	4.1	4.11	4.13	4.13
3.06	3.77	4.24	3.85	3.79	4.23	4.42
3.35	4.28	4.42	4.38	4.71	4.41	4.47
3.52	3.99	4.13	4.32	3.64	4.57	4.32
4.37	4.5	4.62	4.68	4.84	4.41	4.63
3.8	4.57	3.90	4.44	4.49	4.60	4.65
3.06	3.77	4.24	3.85	3.79	4.23	4.42

freq5kg40	freq5kg45	freq5kg50	freq5kg55	freq5kg60	freq5kg65	freq5kg70
4.12	4.17	4.12	4.26	4.26	4.17	4.17
4.9	4.76	4.82	5	4.76	4.77	4.88
4.82	4.55	4.65	4.82	4.82	4.82	4.88
4.72	4.44	4.76	4.71	4.44	4.76	4.83
4.88	4.55	4.35	4.65	4.63	4.26	5
4.45	4.44	4.44	4.49	4.55	4.26	4.45
4.17	4.26	4.21	4.35	4.08	4.21	4.26
4.49	4.44	4.45	4.26	4.35	4.17	4.17
4.08	4.17	4.08	4.26	4.08	4	4.13
4.44	4.72	4.76	4.71	4.44	4.55	4.88
4.55	4.40	4.35	4.65	4.49	4.44	4.44
4.39	4.09	4.66	4.43	4.09	4.55	4.08
4.51	4.52	3.98	4.81	4.75	4.48	4.43
4.33	4.53	4.44	4.08	4.44	4.34	4.44
4.8	4.75	5.12	4.49	4.99	4.7	4.88
4.19	4.15	4.2	4.34	4.07	4.25	4.16
4.19	4.07	4.24	4.16	4.16	4.17	4.08
4.07	3.99	4.25	4.08	3.92	4.09	4.34
4.42	4.48	4.64	4.59	4.34	4.49	4.34
4.15	4.33	3.91	4.52	4.25	4.34	4.21
4.75	4.43	4.64	4.6	4.45	4.61	4.55
4.55	4.49	4.65	4.60	4.60	4.65	4.44
4.07	3.99	4.25	4.08	3.92	4.09	4.34

freq5kg75	freq5kg80	freq5kg85	freq5kg90	freq5kg95	freq5kg100	leng5kg5
4	4.3	4.26	3.92	4.35	3.7	1.46
4.88	5	5	4.65	4.94	4.76	1.31
4.76	4.55	4.76	4.65	4.55	4.30	1.33
4.55	4.65	4.77	4.35	4.88	4.6	1.35
4.52	4.26	4.55	4.88	4.17	4.23	1.41
4.55	4.35	4.55	4.35	4.55	4.12	1.25
4.26	4.26	4.17	4.17	4.17	3.75	1.3
4.55	4.26	4.3	4.17	4.35	4.01	1.22
4.17	4.26	4.08	4.12	4.55	4.08	1.38
4.65	4.35	4.76	4.6	4.55	4.08	1.34
4.65	4.26	4.35	4.65	4.44	3.85	1.34
4.08	5.41	4.17	4.26	4.54	4.1	1.26
4.54	4.45	4.26	4.46	4.34	4.06	1.16
4.44	4.54	4.25	4.26	4.24	4.38	1.11
4.66	5	4.62	5.03	4.75	4.24	0.97
4.17	4.26	4.26	3.39	4.03	3.85	1.31
4.12	4.08	4.12	4.01	3.94	3.75	1.27
3.92	4.09	4.09	4.02	3.97	3.77	1.43
4.55	4.35	4.66	4.23	4.37	4.59	1.31
4.35	4.17	4.27	4.23	4.21	3.48	1.12
4.66	4.37	4.45	4.51	4.46	4.11	0.99
4.55	4.55	4.65	4.60	4.55	4.48	1.06
3.92	4.09	4.09	4.02	3.97	3.77	1.43

leng5kg10	leng5kg15	leng5kg20	leng5kg25	leng5kg30	leng5kg35	leng5kg40
1.67	1.81	1.92	2.03	2.04	2.17	2.24
1.53	1.69	1.73	1.94	2.16	2.01	2.12
1.47	1.66	1.82	1.83	1.9	1.96	1.97
1.55	1.62	1.87	1.94	2.05	2.01	2.09
1.56	1.65	1.75	1.87	2.04	2	1.9
1.54	1.74	1.81	1.93	2.01	2.16	2.17
1.59	1.79	1.95	2.07	2.03	2.24	2.29
1.63	1.7	1.84	1.84	2.05	2.03	2.06
1.79	1.92	2.12	2.16	2.21	2.29	2.38
1.51	1.7	1.91	1.95	2.06	2.01	2.25
1.77	1.77	1.85	1.91	2.06	2.10	2.15
1.12	1.78	1.89	2.09	2.11	1.96	2.19
1.41	1.42	1.73	1.82	2.02	2.02	1.95
1.54	1.72	1.82	1.88	2	1.98	2.06
1.49	1.62	1.78	1.81	1.81	1.99	1.9
1.56	1.64	1.81	1.85	1.96	1.94	2.03
1.48	1.66	1.8	1.88	1.95	2	2.02
1.71	1.69	2	2.14	1.99	1.95	2.16
1.48	1.62	1.76	1.71	1.88	1.92	1.97
1.43	1.62	1.72	1.88	1.93	1.96	2.08
1.42	1.54	1.63	1.63	1.83	1.78	1.77
1.54	1.64	1.66	1.68	1.77	1.86	1.84
1.71	1.69	2	2.14	1.99	1.95	2.16

leng5kg45	leng5kg50	leng5kg55	leng5kg60	leng5kg65	leng5kg70	leng5kg75
2.24	2.29	2.24	2.25	2.3	2.3	2.39
2.22	2.22	2.17	2.29	2.3	2.26	2.26
2.15	1.98	2.07	2.1	2.09	2.06	2.09
2.26	2.13	2.18	2.34	2.19	2.18	2.32
2.09	2.22	2.1	2.15	2.34	1.99	2.22
2.22	2.25	2.25	2.25	2.41	2.32	2.27
2.26	2.3	2.24	2.38	2.3	2.27	2.26
2.13	2.16	2.29	2.25	2.36	2.36	2.16
2.38	2.46	2.38	2.48	2.54	2.46	2.43
2.17	2.18	2.22	2.37	2.32	2.16	2.26
2.27	2.32	2.19	2.27	2.30	2.29	2.18
2.37	2.1	2.24	2.43	2.2	2.46	2.55
2.06	2.37	2.02	2.04	2.18	2.23	2.19
1.99	2.05	2.25	2.07	2.12	2.08	2.08
1.95	1.82	2.09	1.88	2.01	1.94	2.03
2.08	2.08	2.03	2.17	2.1	2.15	2.16
2.11	2.04	2.1	2.1	2.11	2.15	2.13
2.23	2.1	2.21	2.31	2.22	2.09	2.32
1.97	1.92	1.96	2.09	2.03	2.1	2.02
2.03	2.25	1.97	2.09	2.05	2.12	2.05
1.92	1.84	1.88	1.94	1.87	1.89	1.83
1.91	1.94	1.93	2.00	1.93	1.90	1.83
2.23	2.1	2.21	2.31	2.22	2.09	2.32

leng5kg80	leng5kg85	leng5kg90	leng5kg95	leng5kg100	flight5kg5	flight5kg10
2.21	2.22	2.4	2.15	2.51	66.67	95
2.21	2.21	2.38	2.25	2.35	63.33	80
2.16	2.1	2.1	2.1	2.27	90	100
2.27	2.23	2.46	2.21	2.37	70	77.5
2.33	2.16	1.99	2.31	2.24	95	95
2.37	2.27	2.37	2.26	2.49	66.67	87.5
2.26	2.3	2.32	2.32	2.63	103.33	110
2.3	2.26	2.31	2.3	2.5	83.33	95
2.37	2.47	2.43	2.24	2.46	57.5	100
2.4	2.18	2.23	2.24	2.46	76.67	85
2.37	2.29	2.13	2.20	2.50	95	117.5
1.88	2.44	2.38	2.2	2.36	105	95
2.24	2.34	2.22	2.25	2.34	91.67	95
2.04	2.18	2.18	2.19	2.08	111.67	102.5
1.88	2.03	1.85	1.92	2.06	108.25	95
2.11	2.11	2.18	2.18	2.22	106	115
2.16	2.14	2.19	2.22	2.28	180	120
2.23	2.22	2.25	2.24	2.24	129	130
2.11	1.97	2.16	2.06	1.94	126.67	100
2.14	2.09	2.09	2.06	2.41	115.33	112.5
1.94	1.89	1.86	1.86	2	74	107.5
1.85	1.90	1.98	2.14	2.37	96.25	117.5
2.23	2.22	2.25	2.24	2.24	129	130

flight5kg15	flight5kg20	flight5kg25	flight5kg30	flight5kg35	flight5kg40	flight5kg45
105	115	112.5	110	117.5	120	120
92.5	85	102.5	115	100	112.5	115
105	115	112.5	115	117.5	112.5	125
80	97.5	100	105	105	107.5	115
95	105	112.5	125	115	100	112.5
100	107.5	102.5	105	120	115	115
112.5	115	120	110	122.5	125	125
107.5	110	110	120	115	112.5	115
130	130	155	130	125	135	130
92.5	102.5	95	97.5	105	120	117.5
107.5	105	105	115	120	115	122.5
137.5	137.5	145	142.5	125	145	165
95	115	125	125	132.5	110	135
120	107.5	100	120	130	130	135
125	130	130	130	140	137.5	137.5
120	130	130	135	130	137.5	145
132.5	137.5	140	135	140	145	155
120	140	140	130	125	145	150
120	122.5	120	132.5	132.5	135	130
135	142.5	150	122.5	142.5	150	135
115	125	125	135	127.5	120	135
122.5	127.5	125	132.5	137.5	135	135
120	140	140	130	125	145	150

flight5kg50	flight5kg55	flight5kg60	flight5kg65	flight5kg70	flight5kg75	flight5kg80
122.5	120	115	117.5	115	125	112.5
105	110	120	117.5	115	120	110
115	117.5	115	112.5	115	117.5	125
110	115	125	115	115	125	115
125	110	115	125	100	120	125
130	115	115	125	127.5	110	125
130	120	130	130	130	130	127.5
115	120	115	110	130	115	135
140	135	135	145	140	140	140
115	110	120	125	105	117.5	130
135	115	122.5	130	120	130	135
145	147.5	170	145	160	155	155
120	122.5	135	140	145	145	140
132.5	140	130	140	145	125	125
125	147.5	105	140	135	145	130
135	135	145	140	145	142.5	130
145	145	145	155	145	147.5	155
135	150	160	142.5	130	160	150
125	127.5	140	127.5	140	127.5	135
155	125	145	140	140	140	147.5
125	132.5	130	130	135	130	137.5
135	130	145	140	145	140	140
135	150	160	142.5	130	160	150

flight5kg85	flight5kg90	flight5kg95	flight5kg100	contact5kg5	contact5kg10	contact5kg15
100	135	100	145	195	155	142.5
110	125	115	115	171.67	130	112.5
115	125	120	132.5	135	120	117.5
120	130	115	125	183.33	145	130
120	105	135	127.5	148.33	135	130
115	117.5	110	107.5	161.67	137.5	130
125	125	125	150	151.67	130	127.5
127.5	135	145	147.5	163.33	150	127.5
140	137.5	110	137.5	192.5	162.5	115
110	122.5	110	137.5	153.33	130	127.5
125	125	120	150	140	130	125
165	150	140	162.5	165	125	102.5
150	150	140	175	163.33	118.33	95
142.5	145	150	155	145	140	115
150	130	140	155	147.5	105	87.5
135	145	140	160	176.67	135	112.5
145	150	155	170	161.67	122.5	107.5
145	155	157.5	165	165	125	110
120	132.5	135	135	150	127.5	102.5
140	142.5	145	177.5	145	127.5	100
130	130	130	152.5	141.25	107.5	97.5
142.5	140	147.5	150	160	107.5	100
145	155	157.5	165	165	125	110

contact5kg20	contact5kg25	contact5kg30	contact5kg35	contact5kg40	contact5kg45	contact5kg50
130	132.5	125	125	122.5	120	120
110	105	105	100	92.5	95	102.5
105	97.5	100	90	95	95	100
127.5	120	117.5	105	105	110	100
120	112.5	110	107.5	105	107.5	105
115	117.5	115	110	110	110	95
120	115	115	117.5	115	110	107.5
122.5	110	115	110	110	110	110
120	90	110	115	110	110	105
120	120	120	100	105	95	95
115	110	107.5	100	105	105	95
92.5	95	90	85	85	80	70
102.5	90	105	97.5	80	85	80
120	125	112.5	95	100	85	92.5
80	75	77.5	70	72.5	70	85
110	105	102.5	100	100	95	102.5
102.5	100	105	100	92.5	90	90
115	120	105	100	100	100	100
102.5	90	92.5	90	90	92.5	90
85	120	95	87.5	90	97.5	100
87.5	80	90	87.5	90	90	90
90	90	87.5	85	80	82.5	82.5
115	120	105	100	100	100	100

contact5kg55	contact5kg60	contact5kg65	contact5kg70	contact5kg75	contact5kg80	contact5kg85
115	120	122.5	125	125	120	135
90	90	92.5	90	85	90	90
90	92.5	95	90	92.5	95	95
97.5	100	95	92.5	95	100	90
105	102.5	110	100	102.5	110	100
107.5	105	110	97.5	110	105	105
110	115	107.5	105	105	107.5	115
115	115	130	110	105	100	105
100	110	105	102.5	100	95	105
102.5	105	95	100	97.5	100	100
100	100	95	105	85	100	105
80	75	75	85	72.5	80	75
87.5	75	82.5	80	75	85	85
105	95	90	80	100	95	92.5
80	75	70	75	67.5	75	65
95	100	95	95	97.5	105	100
95	95	85	100	95	90	97.5
95	95	102.5	100	95	95	100
90	90	95	90	92.5	95	95
97.5	90	90	97.5	90	92.5	95
85	95	87.5	85	85	92.5	95
85	80	80	75	75	77.5	77.5
95	95	102.5	100	95	95	100

contact5kg90	contact5kg95	contact5kg10(	subjectgroup
120	130	125	1
90	87.5	95	1
90	100	100	1
100	90	92.5	1
100	105	110	1
112.5	110	135	1
115	115	117.5	1
105	85	102.5	1
105	110	107.5	1
95	110	107.5	1
90	105	110	1
85	80	82.5	1
75	92.5	75	2
90	87.5	75	2
70	82.5	80	2
95	100	97.5	2
100	100	100	2
95	97.5	105	2
105	95	95	2
95	95	117.5	2
92.5	95	92.5	2
83.2	85	92.5	2
95	97.5	105	
