# Supplementary material for: Comparison of development of step-kinematics of assisted 60 m sprints with different pulling forces between experienced male and female sprinters
Source: PLoS One. 2021 Jul 27;16(7):e0255302. doi: 10.1371/journal.pone.0255302 (PMC8315524; doi:10.1371/journal.pone.0255302)
Supplement: Attachment — Submitted filename: Review.docx [file pone.0255302.s002.docx]

**GENERAL COMMENTS:**

Author made a good effort in addressing the effects of pulling forces (assisted training) on step-kinematics of male and female sprinters. The paper is generally well written except at some points that I addressed in specific comments.

**SPECIFIC COMMENTS**

**Abstract**

Page 2, Line 40-41 – you state “…which could be a training impulse to move the speed barrier upwards”. This seems to be a bit of an overreach with your results as you did not investigate the effects. Maybe using MAY instead of COULD would solve this.

**Introduction**

Page 4, Line 73 – Suggestion: Omit “all the” before the “studies” - Studies of van den Tillar…

Page 4, Line 77 – To what study do you refer by the sentence “They found that women achieve peak velocity earlier than men and that this was lower than in men”?

Page 4, Line 73-80 – Improve the clarity of this part of the test as it is not clear who did what, and which reference refers which statement.

Page 4, Line 80-83 – I’m not sure what is the purpose of the sentence: “Mero and Komi (9)…”. I does not seem to fit with the previous one or the following one. Try to make this and the following sentence more generic and more connected to the previous text.

Page 5, Line 96-97 – Shouldn’t you add WOULD after the “step velocity”?

Page 5, Line 98 – The sentence: “This higher maximal velocity is the result of longer step length, shorter contact times, and higher step rate as found in earlier studies [9, 10, 16, 17]” does not seem to belong here. This was supposed to be cleared out earlier in Introduction. Here just clearly state your hypotheses.

Page 5, Line 99-102 – You state: “In addition, it was expected that 60 m times in women would decrease relatively more than in men…” Was this a hypothesis as well?. After that, you continue with: “due to the fact that the pulling force was equal for men and women and therefore women experience more pulling force relative to their body mass”. Shouldn’t this be part of the methods/results or the discussion? Also, this could be mentioned earlier in introduction.

**Methods**

***Design***

You did not explain what kind of study design you used.

Page 5, Line 107 – What does “international” refer to? This means that they are competing internationally or they are coming from different countries?

Page 5, Line 109 – I do not think you need to state here which pulley system you used. You will explain it in procedures.

***Participants***

Were there participants younger than 18 years as men age of males was 22 ± 6 years? If yes, how did you provide the consent from them? Could you add age ranges?

What was the length of training history?

What part of the season was it?

What about avoiding other types of training for 48 hours? Also, what did they do prior these 48 hours as this could also be a sufficient time for the acute effects of supercompensation on performance, depending on the part of the season were in.

***Procedures***

General comment: Procedures could be explained in more details. Researchers and trainers may have difficulties replicating, thus reducing the applicability and external validity of this study.

Could you structure this part paragraphs instead of single long paragraph for easier reading, better clarity and understanding?

Although I understand why the individualized warmup was performed, a general information about the structure of warmup would be beneficial for the readers of this manuscript. Like this, it seems that 24 different warmups could be performed without any guidance or without any specificity relative to tasks that participants were warming up for. Given that you emphasize that they used individualized warmup does not make it less important, nor does it explain much.

Did you perform the testing as a training session or it was planned within the athletes’ schedule?

Did participants performed familiarization trials?

What was the rest period between the conditions?

Why the 3, 4, and 5 kg trials were not randomized?

Could you provide the reliability and validity of each device used?

Page 6, Line 129-130 – Add more details on how this works or cite the research that explains the procedure in details.

Page 6, Line 135 – I am not sure if I understand how you measured speed. More details may be needed so the study is replicable to a trainer or researcher less familiar with this device.

**Results**

Page 8, Line 178-181 – This is too long sentence. It would be clearer if you divide it into 2 sentences.

Page 9, Table 1 – This table has not been mentioned in results section, yet it is positioned before the Table 2 that you mentioned.. Indicate significant results in this table, same as you did in other tables. In table title, you used (±SD), but this was not how you used it in the table itself.

Page 10, Line 197 – check for the accuracy of “assistance condition”. Should this be “assisted conditions”?

Page 10, Line 196-200 – This sentence contains too much information. Make it two sentences.

Page 11, Line 218-222 – This should be 2 sentences. It would make it easier to fallow.

Page 11-12, Line 224-225 – “post hoc comparison did not find a significant step frequency difference between conditions in women” – to my understanding, this was already shown in Table 3?

Page 12, Line 226-227 – I suggest replacing “step velocity” with “it”, “more” with “further”, and “increasing” with “larger”.

Page 12, Line 228-230 – This statement should be clearer and more accurate. I’m not so sure what you wanted to say here.

**Discussion**

Page14, Line 280 – Maybe using “compared” instead “then” women would be more appropriate here.

Page 14, Line 280 – Consider replacing “This is explainable” with “This could be partially explained”. Also, this statement should be referenced.

Page 14, Line 282 – You start the sentence with “As maximal stride length correlates very well with step length (r = 0.59–0.70) [19, 23-25]. How does this relate to the previous sentence or even with the continuance of this very sentence? I am not really sure what you wanted to say. You also continue this sentence with “the taller males had 12% longer step length and thereby on average 4 steps less over 60 m”. Does this mean that taller males would have 12% longer step when compared to shorter males? Also, here you used males, while through the text you used man. These are not the synonyms. In my opinion, using sex (females and males) instead of gender (women and men) is more appropriate for this research as you are investigating differences performance produced by two biological sexes. However, I do not know whether this fits journal requirements and policies.

Page 14, Line 277-284 – Consider making this part better connected with the following of this paragraph. Transition from anthropometrics to muscular potential to exert forces should be smoother and better linked. Although you may not have it in your sample, males and females occasionally may be of the same height. However, it does not necessarily mean that they would perform the same (i.e., have the same step length and velocity).

Page 15, Line 208-309 – Consider replacing “mainly caused” by “followed”. How are you sure that the changes in 60-m sprint time were caused by an increase in maximal step velocity and by holding this step velocity longer and not the other way around. By pulling the athletes, you are actually the one increasing their sprinting speed and then you follow what is going on with the kinematics and dynamics. Whether a repeated application of your conditions produce acute increase the maximal step velocity and prolong it so sprinters become faster was not investigated here.

Page 15, Line 310-313 – Make this argument clearer. From the stated here, I am not sure what are you trying to say.

Page 16, Line 334-335 – Should you add “in earlier phases” at the end of this sentence?

Page 16, Line 335-337 – You state: “This was probably caused by the positioning of the athletes as they leaned forwards for producing maximal horizontal forces [1, 31, 32], without breaking the steps”. How does the positioning explain non-significant effects of pulling forces in the early phase of sprint?
