# Supplementary material for: Comparison of development of step-kinematics of assisted 60 m sprints with different pulling forces between experienced male and female sprinters
Source: PLoS One. 2021 Jul 27;16(7):e0255302. doi: 10.1371/journal.pone.0255302 (PMC8315524; doi:10.1371/journal.pone.0255302)
Supplement: Attachment — Submitted filename: comments to the reviewers.docx [file pone.0255302.s004.docx]

Reviewer #1: No review received.

Reviewer #2: Please divide the Introduction into more paragraphs for easier readability.

The discussion of training status, timing of prediction, repetition range (indicative of relative load), set number, and upper or lower body exercise on repetitions in reserve prediction is great. However, there is no mention of the relationship between bar velocity and repetitions in reserve. The closer to task failure a set, the slower the bar velocity will be. Bar velocity would be an objective method of determining repetitions remaining to task failure. A discussion of the relationship between velocity and task failure should be added to this manuscript. See the following recent reviews:

Weakley, Jonathon PhD1,2; Mann, Bryan PhD3; Banyard, Harry PhD4; McLaren, Shaun PhD2,5; Scott, Tannath PhD2,6; Garcia-Ramos, Amador PhD7,8 Velocity-Based Training: From Theory to Application, Strength and Conditioning Journal: April 2021 - Volume 43 - Issue 2 - p 31-49 doi: 10.1519/SSC.0000000000000560.

Dankel SJ, Jessee MB, Mattocks KT, Mouser JG, Counts BR, Buckner SL, Loenneke JP. Training to Fatigue: The Answer for Standardization When Assessing Muscle Hypertrophy? Sports Med. 2017 Jun;47(6):1021-1027. doi: 10.1007/s40279-016-0633-7. PMID: 27677915.

Great amount of work went into this manuscript, even contacting authors of studies to get mean and raw values when not reported--great job!

Very thorough reporting in Results section; sections clearly organized with well done figures--great job!

Again, would like to see the review include bar velocity as a predictor of repetitions in reserve.

Good discussion of the potential implications of underprediction by 1 repetition on neuromuscular adaptations.

Good points on training status and heterogeneity between studies and the lack of clarity between RM and MF as set endpoints.

The "self-fulfilling prophecy" is a huge limitation of these types of studies as you correctly point out. Good that you included the Armes et al. study. Studying this topic is difficult due to this big limitation and not knowing whether a participant is truly pushing themselves to task failure or just doing the exact number of repetitions that they predicted prior to the set. You discussed predicting during the set as a preferred approach which was good. However, I believe that bar velocity could be a better method of creating reliability with the repetitions in reserve method.
